# Stochastic and Statistical Analysis of Cnoidal, Snoidal, Dnoidal, Hyperbolic, Trigonometric and Exponential Wave Solutions of a Coupled Volatility Option-Pricing System

**DOI:** 10.3390/e28030353

**Published:** 2026-03-20

**Authors:** L. M. Abdalgadir, Shabir Ahmad, Bakri Youniso, Khaled Aldwoah

**Affiliations:** 1 Physics Department, College of Science, Imam Mohammad Ibn Saud Islamic University (IMSIU), Riyadh 11623, Saudi Arabia; 2 Department of Mathematics and Physics, University of Campania ‘‘Luigi Vanvitelli’’, 81100 Caserta, Italy; 3 Department of Mathematics, College of Science, King Khalid University, Abha 61421, Saudi Arabia; 4 Department of Mathematics, Faculty of Science, Islamic University of Madinah, Madinah 42351, Saudi Arabia

**Keywords:** white noise, volatility, option-pricing, solitons, statistical measures

## Abstract

We investigate a stochastic coupled nonlinear Schrödinger (Manakov-type) system for option price and volatility wave fields within the Ivancevic adaptive-wave option-pricing paradigm, and derive exact wave families together with statistical diagnostics of the resulting dynamics. This system combines behavioral market effects with classical efficient-market dynamics and incorporates a controlled stochastic volatility component. Randomness in both the option price and volatility is incorporated via white noise, and a system of stochastic partial differential equations (PDEs) is developed that governs the joint evolution of option prices and stock price volatility. We derive advanced solutions of the proposed system using a newly created methodology. The obtained solutions are expressions of cnoidal, snoidal, dnoidal, hyperbolic, trigonometric, and exponential functions. The stochastic dynamical investigation, together with the statistical measures are presented. The autocorrelation function (ACF) of squared returns for the obtained analytical solutions is demonstrated to show distinct differences in second-order temporal dependence, while asymmetries in the temporal evolution of the fluctuations are depicted via leverage correlation (LC). The probability distribution function (PDF) dynamics of the soliton solutions illustrate prominent temporal variability and non-stationary statistical dynamics. Differences in dynamical coupling between the two components of the considered system are presented via phase velocity cross-correlation analysis and are supported by phase difference dynamics visualizations. The strength and structure of coupling between components are displayed via the amplitude cross-correlation function. Mean amplitude dynamics and variance as a function of noise intensity σ, provide a systematic influence of stochastic forcing on their energy and a quantitative measure of stochastic dispersion of soliton solutions. All the results are displayed in 3D and 2D graphs of the stochastics and statistical dynamics of the obtained solutions.

## 1. Introduction

Over the past several decades, PDEs have received special attention from mathematicians due to their vast applications in many branches of science and engineering [1]. One of the most important classes of nonlinear PDEs are integrable systems, which possess exact solutions, Lax pairs, and conservation laws. A basic and well-known integrable equation is the standard KdV equation, used for analysis of shallow water waves [2]. There are many integrable PDEs proposed by researchers in the literature to study different physical phenomena [3,4].

In the context of mathematical finance, the Black–Scholes (BS) equation represents a fundamental parabolic PDE governing the valuation of derivative securities. In 1973, Fischer Black and Myron Scholes originally developed the BS equation and introduced a rigorous analytical structure for option pricing based on rational pricing market assumptions and continuous time stochastic processes [5]. The BS model illustrates the evolution of an option’s price as a function of time and the underlying asset value, with the assumption that the asset process follows a geometric Brownian motion with constant volatility and interest rate. The fundamental BS equation can be written as:

(1) $$B_{t}+\frac{1}{2}Q^{2}x^{2}B_{xx}+\zeta xB_{x}=\zeta B,$$

where B=B(x,t) represents the European call option, the stock price is represented by *x*, the volatility of return is expressed by Q, while ζ denotes the risk-free interest rate. To incorporate both efficient and behavioral efficient marker dynamics, Ivancevic [6] introduced a nonlinear PDE as follows:

(2) $$iR_{t}\left(x,t\right)+\frac{1}{2}QR_{xx}+A\left(\zeta ,y\right)R{\left|R\right|}^{2}=0,$$

where R(x,t) is the option price as a function of the underlying asset and time, and A(ζ,y) defines the adaptive market potential, commonly referred to as the Landau Coefficient. In the simplest non-adaptive case, this coefficient reduces to the constant in interest rate ζ, while in the adaptive case, it relies on a set of tunable parameters $\left\{y_{i}\right\}$. To add controlled stochastic volatility within the adaptive-wave framework [6], the full bidirectional quantum neural computation model for option pricing can be developed as a self-organizing system of two coupled self-focusing nonlinear Shrödinger equations. This model governs the combined evolution of the option price and the stochastic volatility field and is expressed as follows:

(3) $$\begin{array}{ccc} iQ_{t}+\frac{1}{2}Q_{xx}+A\left(\zeta ,y\right)\left({\left|Q\right|}^{2}+{\left|R\right|}^{2}\right)Q & = & 0, \end{array}$$

(4) $$\begin{array}{ccc} iR_{t}+\frac{1}{2}R_{xx}+A\left(\zeta ,y\right)\left({\left|Q\right|}^{2}+{\left|R\right|}^{2}\right)R & = & 0, \end{array}$$

where the volatility wave function and option-price wave function are denoted by Q(x,t) and R(x,t), respectively. For the simplest case A(ζ,y)=ζ, the system (3) is called the Manakov system (MS) [7]. Following Ivancevic’s alternative to the Black–Scholes model, the option price is modeled by a complex-valued wave function B(x,t), whose modulus square ${\left|B\left(x,t\right)\right|}^{2}$ is interpreted as a probability density function (PDF) for option-price dynamics in terms of the underlying price-like variable *x* and time *t*. In the stochastic-volatility setting, the volatility is itself represented by a wave function P(x,t), leading to a coupled NLS (Manakov-type) system for the joint evolution of the volatility wave and the option-price wave. We adopt this modeling viewpoint in the present work [6].

Empirical asset/option time series exhibit stylized facts such as volatility clustering (persistent autocorrelation in squared returns), heavy-tailed return distributions, and leverage-type asymmetry. These features motivate the diagnostic measures. In contrast to classical stochastic-volatility models, such as the Heston diffusion framework (risk-neutral SDE/PDE pricing with calibration), the Ivancevic/Manakov system studied here is a wave-based phenomenological alternative that represents coupled price–volatility dynamics via nonlinear interactions; our focus is on explicit analytical wave families and the resulting stochastic diagnostics rather than on an arbitrage-free calibrated pricing procedure.

Soliton solutions are the most important and interesting feature of integrable systems. Researchers have introduced several methods to derive soliton solutions of integrable systems [8,9,10]. Kumar and Malik recently introduced an efficient analytical method to derive soliton solutions, which are expressed in trigonometric, hyperbolic, and exponential functions [11]. This technique provides a variety of analytical solutions that are mostly non-singular. Therefore, different studies have been conducted using this approach to derive several soliton solutions of nonlinear PDEs [12,13,14]. Soliton solutions of the MS are explored via different analytical methods. Stalin et al. studied non-degenerate soliton solutions of MS corresponding to distinct wave numbers [15]. Chen and Mihalache used a nonrecursive Darboux transformation to construct rogue waves in MS [16]. Some more studies on soliton solutions of MS are provided in [17,18,19].

Apart from this, stochastic differential equations have been used widely in different fields of applied sciences and engineering [20]. SDEs have many applications in physical systems to describe their randomness and stochastic behaviors under noise. In the field of finance and economics, SDEs have been used to study stochastic data and decision-making problems [21]. In the context of mathematical physics, SDEs recently gained much attention to study unpredictable dynamics of wave propagation in physical systems [22,23]. To analyze and present the stochastic behaviors of advanced soliton solutions of the considered MS, we incorporate white noise in the above system as follows:

(5) $$\begin{array}{ccc} idQ+\left[\frac{1}{2}Q_{xx}+\zeta \left({\left|Q\right|}^{2}+{\left|R\right|}^{2}\right)Q\right]dt+\sigma_{1}QdW_{1}\left(t\right) & = & 0, \end{array}$$

(6) $$\begin{array}{ccc} idR+\left[\frac{1}{2}R_{xx}+\zeta \left({\left|Q\right|}^{2}+{\left|R\right|}^{2}\right)R\right]dt+\sigma_{2}RdW_{2}\left(t\right) & = & 0, \end{array}$$

where $W_{1}$ and $W_{2}$ are independent standard Wiener processes. Throughout this work, the stochastic integrals are interpreted in the Itô sense. Furthermore, $\sigma_{1},\sigma_{2}\geq 0$ denote the noise intensities. The noise is therefore multiplicative (state-dependent) and acts as a stochastic phase modulation of the complex fields. The system (5) and (6) has been analyzed in [24] to study dynamical features and some traveling wave solutions in the context of birefringent fibers. In this work, we investigate more advanced soliton solutions with the aid of a newly created technique [11] under noise effects. Additionally, we analyze some statistical measures of the obtained solutions to present different aspects of the fluctuation dynamics of the waves.

## 2. Brief Review of Methodology

To present a general methodology, we consider a generic PDE as follows:

(7) $$P\;\left(F,F_{x},F_{t},F_{xx},F_{tt},F_{xt},F_{xxt},\ldots \right)=0,$$

where F(x,t) is an unknown function of the independent variables *x* and *t*. Following the Kumar–Malik approach [11], we introduce the traveling-wave transformation

(8) $$F\left(x,t\right)=Q\left(\delta \right),\;\delta =\alpha_{1}x+\gamma_{1}t,$$

which reduces Equation (7) to the ordinary differential equation

(9) $$P^{*}\;\left(Q,Q^{\prime },Q^{\prime \prime },Q^{\prime \prime \prime },Q^{⁗},\ldots \right)=0.$$

The solution of Equation (9) is assumed in the polynomial form

(10) $$Q\left(\delta \right)=\sum_{k=0}^{N}\kappa_{k}\;P^{k}\left(\delta \right),$$

where $\kappa_{k}$(k=0,1,2,…,N) are constants to be determined, and the auxiliary function P(δ) satisfies the first-order differential equation

(11) $${\left(P^{\prime }\left(\delta \right)\right)}^{2}=\omega_{0}P^{4}\left(\delta \right)+\omega_{1}P^{3}\left(\delta \right)+\omega_{2}P^{2}\left(\delta \right)+\omega_{3}P\left(\delta \right)+\omega_{4},$$

in which $\omega_{0},\omega_{1},\omega_{2},\omega_{3},\omega_{4}$ are real constants. Substituting Equation (10), together with Equation (11), into Equation (9) yields a polynomial equation in powers of P(δ). Equating the coefficients of each power to zero leads to a system of algebraic equations for the unknown parameters $\zeta_{1},\zeta_{2}$, $\kappa_{k}$(k=0,1,…,N), and $\omega_{i}$(i=0,1,2,3,4). Solving this system allows one to construct exact traveling-wave solutions of the original nonlinear partial differential Equation (7). Depending on the values of the parameters $\omega_{i}$, Equation (34) admits a variety of pathwise exact solutions, which are classified into the following cases.

*Case 1: Jacobi elliptic function solutions*

**Proposition** **1.**  *When*

(12) $$\omega_{3}=\frac{\omega_{1}\left(4\omega_{0}\omega_{2}-\omega_{1}^{2}\right)}{8\omega_{0}^{2}},\;\omega_{4}=0,$$

*Equation* (34) *yields solutions expressed in terms of Jacobi elliptic functions.*

*Subcase 1.1*

*If $\omega_{0}<0$ and $\left(4\omega_{0}\omega_{2}-\omega_{1}^{2}\right)>0$*, *then*

(13) $$\begin{array}{cc} P_{1}\left(\delta \right) & =\frac{-\omega_{1}}{4\omega_{0}}\pm \frac{\omega_{1}}{4\omega_{0}}cn\;\left(\frac{\sqrt{-\omega_{0}\left(4\omega_{0}\omega_{2}-\omega_{1}^{2}\right)}}{2\omega_{0}}\delta ,\frac{\omega_{1}}{2\sqrt{4\omega_{0}\omega_{2}-\omega_{1}^{2}}}\right),\\ P_{2}\left(\delta \right) & =\frac{-\omega_{1}}{4\omega_{0}}\pm \frac{\omega_{1}}{4\omega_{0}}dn\;\left(\frac{\omega_{1}}{4\sqrt{-\omega_{0}}}\delta ,\frac{2\sqrt{4\omega_{0}\omega_{2}-\omega_{1}^{2}}}{\omega_{1}}\right). \end{array}$$

*Subcase 1.2*

*If $\omega_{0}<0$, $\left(4\omega_{0}\omega_{2}-\omega_{1}^{2}\right)<0$, and $\left(16\omega_{0}\omega_{2}-5\omega_{1}^{2}\right)<0$*, *then*

(14) $$\begin{array}{cc} P_{3}\left(\delta \right) & =\frac{-\omega_{1}}{4\omega_{0}}\pm \frac{\sqrt{-\left(16\omega_{0}\omega_{2}-5\omega_{1}^{2}\right)}}{4\omega_{0}}cn\;\left(\frac{\sqrt{\omega_{0}\left(4\omega_{0}\omega_{2}-\omega_{1}^{2}\right)}}{2\omega_{0}}\delta ,\frac{\sqrt{\left(4\omega_{0}\omega_{2}-\omega_{1}^{2}\right)\left(16\omega_{0}\omega_{2}-5\omega_{1}^{2}\right)}}{2\left(4\omega_{0}\omega_{2}-\omega_{1}^{2}\right)}\right),\\ P_{4}\left(\delta \right) & =\frac{-\omega_{1}}{4\omega_{0}}\pm \frac{\sqrt{-\left(16\omega_{0}\omega_{2}-5\omega_{1}^{2}\right)}}{4\omega_{0}}dn\;\left(\frac{\sqrt{\omega_{0}\left(16\omega_{0}\omega_{2}-5\omega_{1}^{2}\right)}}{4\omega_{0}}\delta ,\frac{2\sqrt{\left(4\omega_{0}\omega_{2}-\omega_{1}^{2}\right)\left(16\omega_{0}\omega_{2}-5\omega_{1}^{2}\right)}}{2\left(16\omega_{0}\omega_{2}-5\omega_{1}^{2}\right)}\right). \end{array}$$

*Subcase 1.3*

*If $\omega_{0}<0$, $\left(4\omega_{0}\omega_{2}-\omega_{1}^{2}\right)>0$, and $\left(16\omega_{0}\omega_{2}-5\omega_{1}^{2}\right)<0$*, *then*

(15) $$\begin{array}{cc} P_{5}\left(\delta \right) & =\frac{-\omega_{1}}{4\omega_{0}}\pm \frac{\sqrt{-\left(16\omega_{0}\omega_{2}-5\omega_{1}^{2}\right)}}{4\omega_{0}}nc\;\left(\frac{\sqrt{-\omega_{0}\left(4\omega_{0}\omega_{2}-\omega_{1}^{2}\right)}}{2\omega_{0}}\delta ,\frac{\omega_{1}}{2\left(4\omega_{0}\omega_{2}-\omega_{1}^{2}\right)}\right),\\ P_{6}\left(\delta \right) & =\frac{-\omega_{1}}{4\omega_{0}}\pm \frac{\sqrt{-\left(16\omega_{0}\omega_{2}-5\omega_{1}^{2}\right)}}{4\omega_{0}}nd\;\left(\frac{\omega_{1}}{2\sqrt{-\omega_{0}}}\delta ,\frac{2\sqrt{4\omega_{0}\omega_{2}-\omega_{1}^{2}}}{\omega_{1}}\right). \end{array}$$

*Subcase 1.4*

*If $\omega_{0}\left(4\omega_{0}\omega_{2}-\omega_{1}^{2}\right)>0$ and $\left(16\omega_{0}\omega_{2}-5\omega_{1}^{2}\right)\left(4\omega_{0}\omega_{2}-\omega_{1}^{2}\right)>0$*, *then*

(16) $$\begin{array}{cc} P_{7}\left(\delta \right) & =\frac{-\omega_{1}}{4\omega_{0}}\pm \frac{\omega_{1}}{4\omega_{0}}nc\;\left(\frac{\sqrt{\omega_{0}\left(4\omega_{0}\omega_{2}-\omega_{1}^{2}\right)}}{2\omega_{0}}\delta ,\frac{\sqrt{\left(4\omega_{0}\omega_{2}-\omega_{1}^{2}\right)\left(16\omega_{0}\omega_{2}-5\omega_{1}^{2}\right)}}{2\left(4\omega_{0}\omega_{2}-\omega_{1}^{2}\right)}\right),\\ P_{8}\left(\delta \right) & =\frac{-\omega_{1}}{4\omega_{0}}\pm \frac{\sqrt{-\left(16\omega_{0}\omega_{2}-5\omega_{1}^{2}\right)}}{4\omega_{0}}nd\;\left(\frac{\sqrt{\omega_{0}\left(16\omega_{0}\omega_{2}-5\omega_{1}^{2}\right)}}{4\omega_{0}}\delta ,\frac{2\sqrt{\left(4\omega_{0}\omega_{2}-\omega_{1}^{2}\right)\left(16\omega_{0}\omega_{2}-5\omega_{1}^{2}\right)}}{2\left(16\omega_{0}\omega_{2}-5\omega_{1}^{2}\right)}\right). \end{array}$$

*Subcase 1.5*

*If $\omega_{0}>0$ and $\left(16\omega_{0}\omega_{2}-5\omega_{1}^{2}\right)<0$*, *then*

(17) $$\begin{array}{cc} P_{9}\left(\delta \right) & =\frac{-\omega_{1}}{4\omega_{0}}\pm \frac{\omega_{1}}{4\omega_{0}}ns\;\left(\frac{\omega_{1}}{2\sqrt{\omega_{0}}}\delta ,\frac{\sqrt{-\left(16\omega_{0}\omega_{2}-5\omega_{1}^{2}\right)}}{\omega_{1}}\right),\\ P_{10}\left(\delta \right) & =\frac{-\omega_{1}}{4\omega_{0}}\pm \frac{\sqrt{-\left(16\omega_{0}\omega_{2}-5\omega_{1}^{2}\right)}}{4\omega_{0}}ns\;\left(\frac{\sqrt{-\omega_{0}\left(16\omega_{0}\omega_{2}-5\omega_{1}^{2}\right)}}{4\omega_{0}}\delta ,\frac{\omega_{1}}{\sqrt{-\left(16\omega_{0}\omega_{2}-5\omega_{1}^{2}\right)}}\right),\\ P_{11}\left(\delta \right) & =\frac{-\omega_{1}}{4\omega_{0}}\pm \frac{\sqrt{-\left(16\omega_{0}\omega_{2}-5\omega_{1}^{2}\right)}}{4\omega_{0}}sn\;\left(\frac{\omega_{1}}{2\sqrt{\omega_{0}}}\delta ,\frac{\sqrt{-\left(16\omega_{0}\omega_{2}-5\omega_{1}^{2}\right)}}{\omega_{1}}\right),\\ P_{12}\left(\delta \right) & =\frac{-\omega_{1}}{4\omega_{0}}\pm \frac{\omega_{1}}{4\omega_{0}}sn\;\left(\frac{\sqrt{-\left(16\omega_{0}\omega_{2}-5\omega_{1}^{2}\right)}}{4\omega_{0}}\delta ,\frac{\omega_{1}}{\sqrt{-\left(16\omega_{0}\omega_{2}-5\omega_{1}^{2}\right)}}\right). \end{array}$$

The second argument of the Jacobi elliptic functions represents the elliptic modulus.

*Case 2: Hyperbolic and trigonometric solutions*

**Proposition** **2.**  *When*

(18) $$\omega_{3}=\frac{\omega_{1}\left(4\omega_{0}\omega_{2}-\omega_{1}^{2}\right)}{8\omega_{0}},\;\omega_{4}=\frac{\omega_{1}{\left(16\omega_{0}\omega_{2}-\omega_{1}^{2}\right)}^{2}}{64\omega_{0}^{3}},$$

*Equation* (34) *yields hyperbolic or trigonometric solutions.*

*Subcase 2.1*

*If $\omega_{0}>0$ and $\left(8\omega_{0}\omega_{2}-3\omega_{1}^{2}\right)<0$*, *then*

(19) $$\begin{array}{cc} P_{13}\left(\delta \right) & =\frac{-\omega_{1}}{4\omega_{0}}\pm \frac{\sqrt{-\left(8\omega_{0}\omega_{2}-3\omega_{1}^{2}\right)}}{4\omega_{0}}\tanh\;\left(\frac{\sqrt{-\omega_{0}\left(8\omega_{0}\omega_{2}-3\omega_{1}^{2}\right)}}{4\omega_{1}}\delta \right),\\ P_{14}\left(\delta \right) & =\frac{-\omega_{1}}{4\omega_{0}}\pm \frac{\sqrt{-\left(8\omega_{0}\omega_{2}-3\omega_{1}^{2}\right)}}{4\omega_{0}}\coth\;\left(\frac{\sqrt{-\omega_{0}\left(8\omega_{0}\omega_{2}-3\omega_{1}^{2}\right)}}{4\omega_{1}}\delta \right). \end{array}$$

*Subcase 2.2*

*If $\omega_{0}>0$ and $\left(8\omega_{0}\omega_{2}-3\omega_{1}^{2}\right)>0$*, *then*

(20) $$\begin{array}{cc} P_{15}\left(\delta \right) & =\frac{-\omega_{1}}{4\omega_{0}}\pm \frac{\sqrt{8\omega_{0}\omega_{2}-3\omega_{1}^{2}}}{4\omega_{0}}\tan\;\left(\frac{\sqrt{\omega_{0}\left(8\omega_{0}\omega_{2}-3\omega_{1}^{2}\right)}}{4\omega_{1}}\delta \right),\\ P_{16}\left(\delta \right) & =\frac{-\omega_{1}}{4\omega_{0}}\pm \frac{\sqrt{8\omega_{0}\omega_{2}-3\omega_{1}^{2}}}{4\omega_{0}}\cot\;\left(\frac{\sqrt{\omega_{0}\left(8\omega_{0}\omega_{2}-3\omega_{1}^{2}\right)}}{4\omega_{1}}\delta \right). \end{array}$$

*Case 3: Sech, csch, sec, and csc solutions*

**Proposition** **3.**  *When*

(21) $$\omega_{3}=\frac{\omega_{1}\left(4\omega_{0}\omega_{2}-\omega_{1}^{2}\right)}{8\omega_{0}^{2}},\;\omega_{4}=\frac{\omega_{1}^{2}\left(16\omega_{0}\omega_{2}-5\omega_{1}^{2}\right)}{256\omega_{0}},$$

*the auxiliary equation admits the following solutions.*

*Subcase 3.1*

*If $\omega_{0}<0$ and $\left(8\omega_{0}\omega_{2}-3\omega_{1}^{2}\right)<0$*, *then*

(22) $$P_{17}\left(\delta \right)=\frac{-\omega_{1}}{4\omega_{0}}\pm \frac{\sqrt{-2\left(8\omega_{0}\omega_{2}-3\omega_{1}^{2}\right)}}{4\omega_{0}}\mathrm{sech}\;\left(\frac{\sqrt{2\omega_{0}\left(8\omega_{0}\omega_{2}-3\omega_{1}^{2}\right)}}{4\omega_{1}}\delta \right).$$

*Subcase 3.2*

*If $\omega_{0}>0$ and $\left(8\omega_{0}\omega_{2}-3\omega_{1}^{2}\right)>0$*, *then*

(23) $$P_{18}\left(\delta \right)=\frac{-\omega_{1}}{4\omega_{0}}\pm \frac{\sqrt{2\left(8\omega_{0}\omega_{2}-3\omega_{1}^{2}\right)}}{4\omega_{0}}\mathrm{csch}\;\left(\frac{\sqrt{2\omega_{0}\left(8\omega_{0}\omega_{2}-3\omega_{1}^{2}\right)}}{4\omega_{1}}\delta \right).$$

*Subcase 3.3*

*If $\omega_{0}>0$ and $\left(8\omega_{0}\omega_{2}-3\omega_{1}^{2}\right)<0$*, *then*

(24) $$\begin{array}{cc} P_{19}\left(\delta \right) & =\frac{-\omega_{1}}{4\omega_{0}}\pm \frac{\sqrt{-2\left(8\omega_{0}\omega_{2}-3\omega_{1}^{2}\right)}}{4\omega_{0}}\sec\;\left(\frac{\sqrt{-2\omega_{0}\left(8\omega_{0}\omega_{2}-3\omega_{1}^{2}\right)}}{4\omega_{1}}\delta \right),\\ P_{20}\left(\delta \right) & =\frac{-\omega_{1}}{4\omega_{0}}\pm \frac{\sqrt{-2\left(8\omega_{0}\omega_{2}-3\omega_{1}^{2}\right)}}{4\omega_{0}}\csc\;\left(\frac{\sqrt{-2\omega_{0}\left(8\omega_{0}\omega_{2}-3\omega_{1}^{2}\right)}}{4\omega_{1}}\delta \right). \end{array}$$

*Case 4: Exponential-function solutions*

**Proposition** **4.**  *When*

(25) $$\omega_{1}=\omega_{3}=\omega_{4}=0,\;\omega_{2}>0,$$

*Equation* (34) *yields the exponential-function solution*

(26) $$P_{21}\left(\delta \right)=\frac{4\zeta_{2}\omega_{2}}{4\zeta_{2}^{2}e^{\sqrt{\omega_{2}}\delta }-\omega_{0}\omega_{2}e^{-\sqrt{\omega_{2}}\delta }}.$$

*If $\omega_{0}=-4\zeta_{2}^{2}/\omega_{2}$*, *then*

(27) $$P_{22}\left(\delta \right)=\frac{\omega_{2}}{2\zeta_{2}}\mathrm{sech}\;\left(\sqrt{\omega_{2}}\delta \right),$$

*whereas for $\omega_{0}=4\zeta_{2}^{2}/\omega_{2}$ one obtains*

(28) $$P_{23}\left(\delta \right)=\frac{\omega_{2}}{2\zeta_{2}}\mathrm{csch}\;\left(\sqrt{\omega_{2}}\delta \right).$$

### Stochastic Solutions Using Proposed Method

In this section, we illustrate the reduction of the proposed system of equations to a set of ordinary differential equations (ODEs) with the help of a traveling wave transformation and then using previous developed strategy of solutions to obtain analytical results for the considered system. We use the following transformations:

(29) $$\begin{array}{cc} \; & Q\left(x,t\right)=Q\left(\delta_{1}\right)\exp[\iota \;\left(\delta_{2}+\sigma_{1}W_{1}\left(t\right)-\frac{1}{2}\sigma_{1}^{2}t\right)],\\ & R\left(x,t\right)=R\left(\delta_{1}\right)\exp[\iota \;\left(\delta_{2}+\sigma_{2}W_{2}\left(t\right)-\frac{1}{2}\sigma_{2}^{2}t\right)], \end{array}$$

where $\delta_{1}=\alpha_{1}x+\gamma_{1}t$ and $\delta_{2}=\alpha_{2}x+\gamma_{2}t$. Substituting Equation (29) into Equations (5) and (6), and applying Itô’s formula, the stochastic terms are removed by the exponential gauge factor, yielding the following coupled deterministic ODEs for the profiles *Q* and *R*:

(30) $$\begin{array}{c} \iota \gamma_{2}Q^{\prime }-\gamma_{2}Q+\frac{1}{2}\left(2\iota \alpha_{1}\alpha_{2}Q^{\prime }+\alpha_{1}^{2}Q^{\prime \prime }-\alpha_{2}^{2}Q\right)+\zeta \left(Q^{3}+R^{2}Q\right)=0, \end{array}$$

(31) $$\begin{array}{c} \iota \gamma_{2}R^{\prime }-\gamma_{2}R+\frac{1}{2}\left(2\iota \alpha_{1}\alpha_{2}R^{\prime }+\alpha_{1}^{2}R^{\prime \prime }-\alpha_{2}^{2}R\right)+\zeta \left(R^{3}+Q^{2}R\right)=0. \end{array}$$

The coupled system (30) and (31) is symmetric in the two components: both equations have the same linear dispersive part, and the nonlinearity depends on the rotationally invariant combination $\left(|Q{|}^{2}+|R{|}^{2}\right)$. A standard way to obtain a tractable subclass of exact solutions for Manakov-type systems is to restrict to the invariant manifold

$$M_{\lambda }=\left\{\left(Q,R\right):\;R=\lambda Q,\;\lambda =\mathrm{const}.\right\},$$

which corresponds to polarization-locked (proportional) components. Substituting R=λQ into (30) and (31) yields the same scalar profile equation in both cases (since $R^{2}Q=\lambda^{2}Q^{3}$ and $Q^{2}R=\lambda Q^{3}$), and therefore the reduction is compatible, and both component equations remain satisfied. In the present work, we focus on this proportional-component subclass in order to construct broad families of closed-form solutions; genuinely vectorial solutions with nonparallel components are left for future investigation. To reduce the above coupled system into a single equation, we assume

R=λQ.

Substituting this relation into Equations (30) and (31), we obtain the following nonlinear ODE:

(32) $$\alpha_{1}^{2}Q^{\prime \prime }+\left(\alpha_{2}^{2}+2\gamma_{2}\right)Q-B\left(1+\lambda^{2}\right)Q^{3}=0,$$

where B=2ζ. Since the homogeneous balance principle k=1, according to the Kumar–Malik methodology, we assume the solution in the form

(33) $$Q\left(\delta_{1}\right)=\kappa_{0}+\kappa_{1}P\left(\delta_{1}\right),$$

where the auxiliary function $P(\delta_{1})$ satisfies the first-order ordinary differential equation

(34) $$P^{\prime }\left(\delta_{1}\right)=\sqrt{\omega_{0}P^{4}\left(\delta_{1}\right)+\omega_{1}P^{3}\left(\delta_{1}\right)+\omega_{2}P^{2}\left(\delta_{1}\right)+\omega_{3}P\left(\delta_{1}\right)+\omega_{4}}.$$

Substituting Equation (33) into Equation (9) and equating the coefficients of like powers of $P(\delta_{1})$, we obtain the following nontrivial parameter relations:

(35) $$\begin{array}{cc} \omega_{0} & =\frac{B\kappa_{1}^{2}\left(1+\lambda^{2}\right)}{\alpha_{1}^{2}}, \end{array}$$

(36) $$\begin{array}{cc} \omega_{1} & =\frac{4B\kappa_{0}\kappa_{1}\left(1+\lambda^{2}\right)}{\alpha_{1}^{2}}, \end{array}$$

(37) $$\begin{array}{cc} \omega_{2} & =\frac{-\alpha_{2}^{2}-2\gamma_{2}+6B\kappa_{0}^{2}\left(1+\lambda^{2}\right)}{\alpha_{1}^{2}}, \end{array}$$

(38) $$\begin{array}{cc} \omega_{3} & =-\frac{2\left(\alpha_{2}^{2}\kappa_{0}-2B\kappa_{0}^{3}\left(1+\lambda^{2}\right)+2\gamma_{2}\kappa_{0}\right)}{\alpha_{1}^{2}\kappa_{1}},\;\omega_{4}\to \omega_{4}. \end{array}$$

Putting some constraint conditions on the above parameters, the following solutions are obtained:

*Case 1: Jacobi elliptic function solutions*

When

(39) $$\omega_{3}=\frac{\omega_{1}\left(4\omega_{0}\omega_{2}-\omega_{1}^{2}\right)}{8\omega_{0}^{2}},\;\omega_{4}=0,$$

Equation (34) yields solutions expressed in terms of Jacobi elliptic functions.

Subcase 1.4

If $\omega_{0}\left(4\omega_{0}\omega_{2}-\omega_{1}^{2}\right)>0$ and $\left(16\omega_{0}\omega_{2}-5\omega_{1}^{2}\right)\left(4\omega_{0}\omega_{2}-\omega_{1}^{2}\right)>0$. Using our obtained parameters’ values, the above conditions are satisfied if the following conditions hold:

$$\omega_{0}\left(4\omega_{0}\omega_{2}-\omega_{1}^{2}\right)>0\;⟺\;2B\kappa_{0}^{2}\left(1+\lambda^{2}\right)>\alpha_{2}^{2}+2\gamma_{2}.$$

Moreover,

$$\left(16\omega_{0}\omega_{2}-5\omega_{1}^{2}\right)\left(4\omega_{0}\omega_{2}-\omega_{1}^{2}\right)>0\;⟺\;B\kappa_{0}^{2}\left(1+\lambda^{2}\right)>\alpha_{2}^{2}+2\gamma_{2}.$$

Therefore, both inequalities hold if and only if

$$B\kappa_{0}^{2}\left(1+\lambda^{2}\right)>\alpha_{2}^{2}+2\gamma_{2},\;\alpha_{1}\neq 0,\;\kappa_{1}\neq 0.$$

Then, in this case, the corresponding final stochastic solutions of the coupled system are given as follows.

(40) $$\begin{array}{cc} Q_{14a}\left(x,t\right)\; & =\left[\kappa_{0}+\kappa_{1}\left(\frac{-\omega_{1}}{4\omega_{0}}\pm \frac{\omega_{1}}{4\omega_{0}}nc\;\left(\frac{\sqrt{\omega_{0}\left(4\omega_{0}\omega_{2}-\omega_{1}^{2}\right)}}{2\omega_{0}}\delta_{1},\frac{\sqrt{\left(4\omega_{0}\omega_{2}-\omega_{1}^{2}\right)\left(16\omega_{0}\omega_{2}-5\omega_{1}^{2}\right)}}{2\left(4\omega_{0}\omega_{2}-\omega_{1}^{2}\right)}\right)\right)\right]\\ \; & \;\times \exp[\iota \;\left(\left(\alpha_{2}x+\gamma_{2}t\right)+\sigma_{1}\left(W\left(t\right)-\frac{1}{2}t\right)\right)],\\ R_{14a}\left(x,t\right)\; & =\lambda \left[\kappa_{0}+\kappa_{1}\left(\frac{-\omega_{1}}{4\omega_{0}}\pm \frac{\omega_{1}}{4\omega_{0}}nc\;\left(\frac{\sqrt{\omega_{0}\left(4\omega_{0}\omega_{2}-\omega_{1}^{2}\right)}}{2\omega_{0}}\delta_{1},\frac{\sqrt{\left(4\omega_{0}\omega_{2}-\omega_{1}^{2}\right)\left(16\omega_{0}\omega_{2}-5\omega_{1}^{2}\right)}}{2\left(4\omega_{0}\omega_{2}-\omega_{1}^{2}\right)}\right)\right)\right]\\ \; & \;\times \exp[\iota \;\left(\left(\alpha_{2}x+\gamma_{2}t\right)+\sigma_{2}\left(W\left(t\right)-\frac{1}{2}t\right)\right)]. \end{array}$$

(41) $$\begin{array}{cc} Q_{14b}\left(x,t\right)\; & =\left[\kappa_{0}+\kappa_{1}\left(-\frac{\omega_{1}}{4\omega_{0}}\pm \frac{\sqrt{-\left(16\omega_{0}\omega_{2}-5\omega_{1}^{2}\right)}}{4\omega_{0}}\;nd\;\left(\Theta_{14a},m_{14}\right)\right)\right]\\ \; & \;\times \exp[\iota \;\left(\left(\alpha_{2}x+\gamma_{2}t\right)+\sigma_{1}\left(W\left(t\right)-\frac{1}{2}t\right)\right)],\\ R_{14b}\left(x,t\right)\; & =\lambda \left[\kappa_{0}+\kappa_{1}\left(-\frac{\omega_{1}}{4\omega_{0}}\pm \frac{\sqrt{-\left(16\omega_{0}\omega_{2}-5\omega_{1}^{2}\right)}}{4\omega_{0}}\;nd\;\left(\Theta_{14a},m_{14}\right)\right)\right]\\ \; & \;\times \exp[\iota \;\left(\left(\alpha_{2}x+\gamma_{2}t\right)+\sigma_{2}\left(W\left(t\right)-\frac{1}{2}t\right)\right)]. \end{array}$$

where

$$\begin{array}{cc} \Theta_{14a} & =\frac{\sqrt{\omega_{0}\left(16\omega_{0}\omega_{2}-5\omega_{1}^{2}\right)}}{4\omega_{0}}\delta_{1},\\ m_{14} & =\frac{\sqrt{\left(4\omega_{0}\omega_{2}-\omega_{1}^{2}\right)\left(16\omega_{0}\omega_{2}-5\omega_{1}^{2}\right)}}{16\omega_{0}\omega_{2}-5\omega_{1}^{2}}. \end{array}$$

Subcase 1.5

If $\omega_{0}>0$ and $\left(16\omega_{0}\omega_{2}-5\omega_{1}^{2}\right)<0$. From the obtained parameters for the solutions, the above inequalities hold if the following conditions are satisfied: From the definitions,

$$\omega_{0}=\frac{B\kappa_{1}^{2}\left(1+\lambda^{2}\right)}{\alpha_{1}^{2}},$$

hence,

$$\omega_{0}>0\;⟺\;B>0\;\left(\alpha_{1}\neq 0,\;\kappa_{1}\neq 0\right).$$

Moreover,

$$16\omega_{0}\omega_{2}-5\omega_{1}^{2}=\frac{16B\kappa_{1}^{2}\left(1+\lambda^{2}\right)}{\alpha_{1}^{4}}\left(B\kappa_{0}^{2}\left(1+\lambda^{2}\right)-\alpha_{2}^{2}-2\gamma_{2}\right).$$

Since the prefactor is positive for B>0, one has

$$16\omega_{0}\omega_{2}-5\omega_{1}^{2}<0\;⟺\;B\kappa_{0}^{2}\left(1+\lambda^{2}\right)<\alpha_{2}^{2}+2\gamma_{2}.$$

Therefore, the conditions

$$\omega_{0}>0,\;16\omega_{0}\omega_{2}-5\omega_{1}^{2}<0,$$

are equivalent to

(42) $$B>0,\;B\kappa_{0}^{2}\left(1+\lambda^{2}\right)<\alpha_{2}^{2}+2\gamma_{2},\;\alpha_{1}\neq 0,\;\kappa_{1}\neq 0.$$

Then, additional solutions of the proposed model are as follows:

(43) $$\begin{array}{cc} Q_{15a}\left(x,t\right)\; & =\left[\kappa_{0}+\kappa_{1}\left(-\frac{\omega_{1}}{4\omega_{0}}\pm \frac{\omega_{1}}{4\omega_{0}}ns\;\left(\frac{\omega_{1}}{2\sqrt{\omega_{0}}}\;\delta_{1},\frac{\sqrt{-\left(16\omega_{0}\omega_{2}-5\omega_{1}^{2}\right)}}{\omega_{1}}\right)\right)\right]\\ \; & \;\times \exp[\iota \;\left(\left(\alpha_{2}x+\gamma_{2}t\right)+\sigma_{1}\left(W\left(t\right)-\frac{1}{2}t\right)\right)],\\ R_{15a}\left(x,t\right)\; & =\lambda \left[\kappa_{0}+\kappa_{1}\left(-\frac{\omega_{1}}{4\omega_{0}}\pm \frac{\omega_{1}}{4\omega_{0}}ns\;\left(\frac{\omega_{1}}{2\sqrt{\omega_{0}}}\;\delta_{1},\frac{\sqrt{-\left(16\omega_{0}\omega_{2}-5\omega_{1}^{2}\right)}}{\omega_{1}}\right)\right)\right]\\ \; & \;\times \exp[\iota \;\left(\left(\alpha_{2}x+\gamma_{2}t\right)+\sigma_{2}\left(W\left(t\right)-\frac{1}{2}t\right)\right)]. \end{array}$$

(44) $$\begin{array}{cc} Q_{15b}\left(x,t\right)\; & =[\kappa_{0}+\kappa_{1}(-\frac{\omega_{1}}{4\omega_{0}}\pm \frac{\sqrt{-\left(16\omega_{0}\omega_{2}-5\omega_{1}^{2}\right)}}{4\omega_{0}}\\ \; & \;\;\;\times ns\;\left(\frac{\sqrt{-\omega_{0}\left(16\omega_{0}\omega_{2}-5\omega_{1}^{2}\right)}}{4\omega_{0}}\;\delta_{1},\frac{\omega_{1}}{\sqrt{-\left(16\omega_{0}\omega_{2}-5\omega_{1}^{2}\right)}}\right))]\\ \; & \;\times \exp[\iota \;\left(\left(\alpha_{2}x+\gamma_{2}t\right)+\sigma_{1}\left(W\left(t\right)-\frac{1}{2}t\right)\right)],\\ R_{15b}\left(x,t\right)\; & =\lambda [\kappa_{0}+\kappa_{1}(-\frac{\omega_{1}}{4\omega_{0}}\pm \frac{\sqrt{-\left(16\omega_{0}\omega_{2}-5\omega_{1}^{2}\right)}}{4\omega_{0}}\\ \; & \;\;\;\times ns\;\left(\frac{\sqrt{-\omega_{0}\left(16\omega_{0}\omega_{2}-5\omega_{1}^{2}\right)}}{4\omega_{0}}\;\delta_{1},\frac{\omega_{1}}{\sqrt{-\left(16\omega_{0}\omega_{2}-5\omega_{1}^{2}\right)}}\right))]\\ \; & \;\times \exp[\iota \;\left(\left(\alpha_{2}x+\gamma_{2}t\right)+\sigma_{2}\left(W\left(t\right)-\frac{1}{2}t\right)\right)]. \end{array}$$

(45) $$\begin{array}{cc} Q_{15c}\left(x,t\right)\; & =[\kappa_{0}+\kappa_{1}(-\frac{\omega_{1}}{4\omega_{0}}\pm \frac{\sqrt{-\left(16\omega_{0}\omega_{2}-5\omega_{1}^{2}\right)}}{4\omega_{0}}\\ \; & \;\;\;\times sn\;\left(\frac{\omega_{1}}{2\sqrt{\omega_{0}}}\;\delta_{1},\frac{\sqrt{-\left(16\omega_{0}\omega_{2}-5\omega_{1}^{2}\right)}}{\omega_{1}}\right))]\\ \; & \;\times \exp[\iota \;\left(\left(\alpha_{2}x+\gamma_{2}t\right)+\sigma_{1}\left(W\left(t\right)-\frac{1}{2}t\right)\right)],\\ R_{15c}\left(x,t\right)\; & =\lambda [\kappa_{0}+\kappa_{1}(-\frac{\omega_{1}}{4\omega_{0}}\pm \frac{\sqrt{-\left(16\omega_{0}\omega_{2}-5\omega_{1}^{2}\right)}}{4\omega_{0}}\\ \; & \;\;\;\times sn\;\left(\frac{\omega_{1}}{2\sqrt{\omega_{0}}}\;\delta_{1},\frac{\sqrt{-\left(16\omega_{0}\omega_{2}-5\omega_{1}^{2}\right)}}{\omega_{1}}\right))]\\ \; & \;\times \exp[\iota \;\left(\left(\alpha_{2}x+\gamma_{2}t\right)+\sigma_{2}\left(W\left(t\right)-\frac{1}{2}t\right)\right)]. \end{array}$$

(46) $$\begin{array}{cc} Q_{15d}\left(x,t\right)\; & =\left[\kappa_{0}+\kappa_{1}\left(-\frac{\omega_{1}}{4\omega_{0}}\pm \frac{\omega_{1}}{4\omega_{0}}sn\;\left(\frac{\sqrt{-\left(16\omega_{0}\omega_{2}-5\omega_{1}^{2}\right)}}{4\omega_{0}}\;\delta_{1},\frac{\omega_{1}}{\sqrt{-\left(16\omega_{0}\omega_{2}-5\omega_{1}^{2}\right)}}\right)\right)\right]\\ \; & \;\times \exp[\iota \;\left(\left(\alpha_{2}x+\gamma_{2}t\right)+\sigma_{1}\left(W\left(t\right)-\frac{1}{2}t\right)\right)],\\ R_{15d}\left(x,t\right)\; & =\lambda \left[\kappa_{0}+\kappa_{1}\left(-\frac{\omega_{1}}{4\omega_{0}}\pm \frac{\omega_{1}}{4\omega_{0}}sn\;\left(\frac{\sqrt{-\left(16\omega_{0}\omega_{2}-5\omega_{1}^{2}\right)}}{4\omega_{0}}\;\delta_{1},\frac{\omega_{1}}{\sqrt{-\left(16\omega_{0}\omega_{2}-5\omega_{1}^{2}\right)}}\right)\right)\right]\\ \; & \;\times \exp\exp[\iota \;\left(\left(\alpha_{2}x+\gamma_{2}t\right)+\sigma_{2}\left(W\left(t\right)-\frac{1}{2}t\right)\right)]. \end{array}$$

The second argument of the Jacobi elliptic functions represents the elliptic modulus.

*Case 2: Hyperbolic and trigonometric solutions*

When

(47) $$\omega_{3}=\frac{\omega_{1}\left(4\omega_{0}\omega_{2}-\omega_{1}^{2}\right)}{8\omega_{0}},\;\omega_{4}=\frac{\omega_{1}{\left(16\omega_{0}\omega_{2}-\omega_{1}^{2}\right)}^{2}}{64\omega_{0}^{3}},$$

Equation (34) yields hyperbolic or trigonometric solutions.

Subcase 2.1

If $\omega_{0}>0$ and $\left(8\omega_{0}\omega_{2}-3\omega_{1}^{2}\right)<0$. From

$$\omega_{0}=\frac{B\kappa_{1}^{2}\left(1+\lambda^{2}\right)}{\alpha_{1}^{2}},$$

one has

$$\omega_{0}>0\;⟺\;B>0\;\left(\alpha_{1}\neq 0,\;\kappa_{1}\neq 0\right).$$

Moreover,

$$8\omega_{0}\omega_{2}-3\omega_{1}^{2}=\frac{8B\kappa_{1}^{2}\left(1+\lambda^{2}\right)}{\alpha_{1}^{4}}\left(3B\kappa_{0}^{2}\left(1+\lambda^{2}\right)-\alpha_{2}^{2}-2\gamma_{2}\right).$$

Since the prefactor is positive for B>0, it follows that

$$8\omega_{0}\omega_{2}-3\omega_{1}^{2}<0\;⟺\;3B\kappa_{0}^{2}\left(1+\lambda^{2}\right)<\alpha_{2}^{2}+2\gamma_{2}.$$

Therefore,

$$\omega_{0}>0,\;8\omega_{0}\omega_{2}-3\omega_{1}^{2}<0\;⟺\;B>0,\;3B\kappa_{0}^{2}\left(1+\lambda^{2}\right)<\alpha_{2}^{2}+2\gamma_{2},\;\alpha_{1}\neq 0,\;\kappa_{1}\neq 0.$$

Then, the following solutions are obtained from the proposed model:

(48) $$\begin{array}{cc} Q_{21a}\left(x,t\right)\; & =\left[\kappa_{0}+\kappa_{1}\left(\frac{-\omega_{1}}{4\omega_{0}}\pm \frac{\sqrt{-\left(8\omega_{0}\omega_{2}-3\omega_{1}^{2}\right)}}{4\omega_{0}}\tanh\;\left(\frac{\sqrt{-\omega_{0}\left(8\omega_{0}\omega_{2}-3\omega_{1}^{2}\right)}}{4\omega_{1}}\delta_{1}\right)\right)\right]\\ \; & \;\times \exp[\iota \;\left(\left(\alpha_{2}x+\gamma_{2}t\right)+\sigma_{1}\left(W\left(t\right)-\frac{1}{2}t\right)\right)],\\ R_{21a}\left(x,t\right)\; & =\lambda \left[\kappa_{0}+\kappa_{1}\left(\frac{-\omega_{1}}{4\omega_{0}}\pm \frac{\sqrt{-\left(8\omega_{0}\omega_{2}-3\omega_{1}^{2}\right)}}{4\omega_{0}}\tanh\;\left(\frac{\sqrt{-\omega_{0}\left(8\omega_{0}\omega_{2}-3\omega_{1}^{2}\right)}}{4\omega_{1}}\delta_{1}\right)\right)\right]\\ \; & \;\times \exp[\iota \;\left(\iota \left(\alpha_{2}x+\gamma_{2}t\right)+\sigma_{2}\left(W\left(t\right)-\frac{1}{2}t\right)\right)]. \end{array}$$

Solution associated with $P_{21b}\left(\delta \right)$.

(49) $$\begin{array}{cc} Q_{21b}\left(x,t\right)\; & =\left[\kappa_{0}+\kappa_{1}\left(\frac{-\omega_{1}}{4\omega_{0}}\pm \frac{\sqrt{-\left(8\omega_{0}\omega_{2}-3\omega_{1}^{2}\right)}}{4\omega_{0}}\coth\;\left(\frac{\sqrt{-\omega_{0}\left(8\omega_{0}\omega_{2}-3\omega_{1}^{2}\right)}}{4\omega_{1}}\delta_{1}\right)\right)\right]\\ \; & \;\times \exp[\iota \;\left(\iota \left(\alpha_{2}x+\gamma_{2}t\right)+\sigma_{1}\left(W\left(t\right)-\frac{1}{2}t\right)\right)],\\ R_{21b}\left(x,t\right)\; & =\lambda \left[\kappa_{0}+\kappa_{1}\left(\frac{-\omega_{1}}{4\omega_{0}}\pm \frac{\sqrt{-\left(8\omega_{0}\omega_{2}-3\omega_{1}^{2}\right)}}{4\omega_{0}}\coth\;\left(\frac{\sqrt{-\omega_{0}\left(8\omega_{0}\omega_{2}-3\omega_{1}^{2}\right)}}{4\omega_{1}}\delta_{1}\right)\right)\right]\\ \; & \;\times \exp[\iota \;\left(\iota \left(\alpha_{2}x+\gamma_{2}t\right)+\sigma_{2}\left(W\left(t\right)-\frac{1}{2}t\right)\right)]. \end{array}$$

Subcase 2.2

If $\omega_{0}>0$ and $\left(8\omega_{0}\omega_{2}-3\omega_{1}^{2}\right)>0$. From the obtained parameters’ values, the above inequalities hold if the following conditions are satisfied: From

$$\omega_{0}=\frac{B\kappa_{1}^{2}\left(1+\lambda^{2}\right)}{\alpha_{1}^{2}},$$

one has

$$\omega_{0}>0\;⟺\;B>0\;\left(\alpha_{1}\neq 0,\;\kappa_{1}\neq 0\right).$$

Moreover,

$$8\omega_{0}\omega_{2}-3\omega_{1}^{2}=\frac{8B\kappa_{1}^{2}\left(1+\lambda^{2}\right)}{\alpha_{1}^{4}}\left(3B\kappa_{0}^{2}\left(1+\lambda^{2}\right)-\alpha_{2}^{2}-2\gamma_{2}\right).$$

As we know that the pre-factor is positive for B>0, it follows that

$$8\omega_{0}\omega_{2}-3\omega_{1}^{2}>0\;⟺\;3B\kappa_{0}^{2}\left(1+\lambda^{2}\right)>\alpha_{2}^{2}+2\gamma_{2}.$$

Therefore,

$$\omega_{0}>0,\;8\omega_{0}\omega_{2}-3\omega_{1}^{2}>0\;⟺\;B>0,\;3B\kappa_{0}^{2}\left(1+\lambda^{2}\right)>\alpha_{2}^{2}+2\gamma_{2},\;\alpha_{1}\neq 0,\;\kappa_{1}\neq 0.$$

Then, the following solutions are obtained:

(50) $$\begin{array}{cc} Q_{22a}\left(x,t\right)\; & =\left[\kappa_{0}+\kappa_{1}\left(-\frac{\omega_{1}}{4\omega_{0}}\pm \frac{\sqrt{8\omega_{0}\omega_{2}-3\omega_{1}^{2}}}{4\omega_{0}}\tan\left(\frac{\sqrt{\omega_{0}\left(8\omega_{0}\omega_{2}-3\omega_{1}^{2}\right)}}{4\omega_{1}}\;\delta_{1}\right)\right)\right]\\ \; & \;\times \exp[\iota \;\left(\left(\alpha_{2}x+\gamma_{2}t\right)+\sigma_{1}\left(W\left(t\right)-\frac{1}{2}t\right)\right)],\\ R_{22a}\left(x,t\right)\; & =\lambda \left[\kappa_{0}+\kappa_{1}\left(-\frac{\omega_{1}}{4\omega_{0}}\pm \frac{\sqrt{8\omega_{0}\omega_{2}-3\omega_{1}^{2}}}{4\omega_{0}}\tan\left(\frac{\sqrt{\omega_{0}\left(8\omega_{0}\omega_{2}-3\omega_{1}^{2}\right)}}{4\omega_{1}}\;\delta_{1}\right)\right)\right]\\ \; & \;\times \exp[\iota \left(\iota \left(\alpha_{2}x+\gamma_{2}t\right)+\sigma_{2}\left(W\left(t\right)-\frac{1}{2}t\right)\right)]. \end{array}$$

(51) $$\begin{array}{cc} Q_{22b}\left(x,t\right)\; & =\left[\kappa_{0}+\kappa_{1}\left(-\frac{\omega_{1}}{4\omega_{0}}\pm \frac{\sqrt{8\omega_{0}\omega_{2}-3\omega_{1}^{2}}}{4\omega_{0}}\cot\left(\frac{\sqrt{\omega_{0}\left(8\omega_{0}\omega_{2}-3\omega_{1}^{2}\right)}}{4\omega_{1}}\;\delta_{1}\right)\right)\right]\\ \; & \;\times \exp[\iota \;\left(\iota \left(\alpha_{2}x+\gamma_{2}t\right)+\sigma_{1}\left(W\left(t\right)-\frac{1}{2}t\right)\right)],\\ R_{22b}\left(x,t\right)\; & =\lambda \left[\kappa_{0}+\kappa_{1}\left(-\frac{\omega_{1}}{4\omega_{0}}\pm \frac{\sqrt{8\omega_{0}\omega_{2}-3\omega_{1}^{2}}}{4\omega_{0}}\cot\left(\frac{\sqrt{\omega_{0}\left(8\omega_{0}\omega_{2}-3\omega_{1}^{2}\right)}}{4\omega_{1}}\;\delta_{1}\right)\right)\right]\\ \; & \;\times \exp[\iota \;\left(\iota \left(\alpha_{2}x+\gamma_{2}t\right)+\sigma_{2}\left(W\left(t\right)-\frac{1}{2}t\right)\right)]. \end{array}$$

## 3. Stochastic Behavioral Diagnostics and Statistical Measures

This part of the manuscript presents the stochastic dynamical diagnostics together with the statistical measures. First, to study the stochastic volatility dynamics, we extract the time series at a fixed spatial location $x=x_{0}$. This reduces the spatio-temporal stochastic fields to temporal processes only. We defined the amplitude process by

(52) $$V\left(t\right)=|Q\left(x_{0},t\right)|\;\mathrm{or}\;V\left(t\right)=\left|R\left(x_{0},t\right)\right|,$$

which represents the local energy envelope of the component. As we know that noise is induced multiplicatively, the amplitude evolves as a positive random process whose fluctuations encode volatility-like behavior. Discrete logarithmic returns are constructed from the amplitude time series as

(53) $$r\left(t_{k}\right)=\log V\left(t_{k+1}\right)-\log V\left(t_{k}\right),$$

which measure relative changes in amplitude over successive time steps. This criterion removes slow trends and highlights isolated bursts induced by the noise. This makes it suitable for statistical analysis of volatility. Volatility clustering is quantified using the autocorrelation function of squared returns,

(54) $$\mathrm{ACF}\left(\ell \right)=\frac{\mathrm{Cov}\;\left(r^{2}\left(t\right),\;r^{2}\left(t+\ell \right)\right)}{\mathrm{Var}\;\left(r^{2}\left(t\right)\right)},$$

where *ℓ* denotes the time lag. A slow decay of ACF(ℓ) indicates persistence in the magnitude of fluctuations, meaning large-amplitude variations tend to cluster in time, even when returns themselves remain weakly correlated.

The leverage effect describes the asymmetry between past fluctuations and future variability, which is measured by

(55) $$L\left(\tau \right)=\mathrm{Corr}\;\left(r(t),\;|r(t+\tau )|\right),$$

where τ>0 is the lag. We report L(τ) as a diagnostic of asymmetry in the model-generated return series; under Gaussian multiplicative forcing, a persistent negative leverage pattern is not guaranteed for all parameter sets; thus, the figures illustrate the behavior observed for the selected solutions and parameters.

The phase dynamics are extracted from complex-valued fields by defining the following:

(56) $$\phi_{Q}\left(t\right)=\arg Q\left(x_{0},t\right),\;\phi_{R}\left(t\right)=\arg R\left(x_{0},t\right).$$

The relative phase evolution is characterized by the phase difference

(57) $$\Delta \phi \left(t\right)=\phi_{Q}\left(t\right)-\phi_{R}\left(t\right),$$

which quantifies synchronization between the two coupled components. Persistent boundedness of the Δϕ(t) presents phase locking, whereas isolated jumps correspond to the phase slip events. The degree of the phase synchronization is measured by the phase locking value (PLV),

(58) $$\mathrm{PLV}=\left|\langle e^{i\Delta \phi (t)}\rangle \right|,$$

where 〈·〉 denotes temporal averaging. Instantaneous phase velocities, defined by ${\dot{\phi }}_{Q}\left(t\right)$ and ${\dot{\phi }}_{R}\left(t\right)$, are used to compute the phase velocity cross-correlation.

The stochastic model (5) and (6) is defined on a filtered probability space $\left(\Omega ,F,{\left\{F_{t}\right\}}_{t\geq 0},P\right)$ that supports independent standard Wiener processes $W_{1}\left(t\right)$ and $W_{2}\left(t\right)$. Throughout, E[·] denotes expectation with respect to P (ensemble averaging over noise realizations). When a time average along a single realization is used, we denote it by ${\langle f\left(t\right)\rangle }_{t}$, i.e.,

$${\langle f\left(t\right)\rangle }_{t}:=\frac{1}{T}\int_{0}^{T}f\left(t\right)\;dt,$$

or its discrete-time analog on the sampled grid. Ensemble expectations are approximated numerically by Monte-Carlo averaging, $E\left[f\right]\approx \frac{1}{N_{\mathrm{MC}}}\sum_{n=1}^{N_{\mathrm{MC}}}f^{\left(n\right)}$, where $f^{\left(n\right)}$ is computed from the *n*-th independent noise realization. Unless stated otherwise, ${\langle \cdot \rangle }_{t}$ refers to time averaging and E[·] refers to ensemble averaging. The noise-induced transitions are characterized through the low-order statistical moments of the amplitude process. The mean amplitude,

(59) μ(σ)=E[V(t)],

quantifies noise-driven shifts in average energy level, while the variance,

(60) $$\mathrm{Var}\left(\sigma \right)=E[{\left(V\left(t\right)-\mu \right)}^{2}],$$

measures the growth of fluctuations and dispersion with increasing noise intensity σ.

**Remark** **1.**  *The deterministic traveling-wave cores provide structured baseline dynamics, whereas the volatility clustering (ACF of squared returns) and leverage-type correlations reported here arise from the stochastic multiplicative modulation induced by the Wiener-driven factors in the analytical solutions. In other words, these econometric-style diagnostics are evaluated on the stochastic sample-path returns generated by the model, not on the deterministic cores alone.*

## 4. Simulations and Discussion

**Figure 1:**
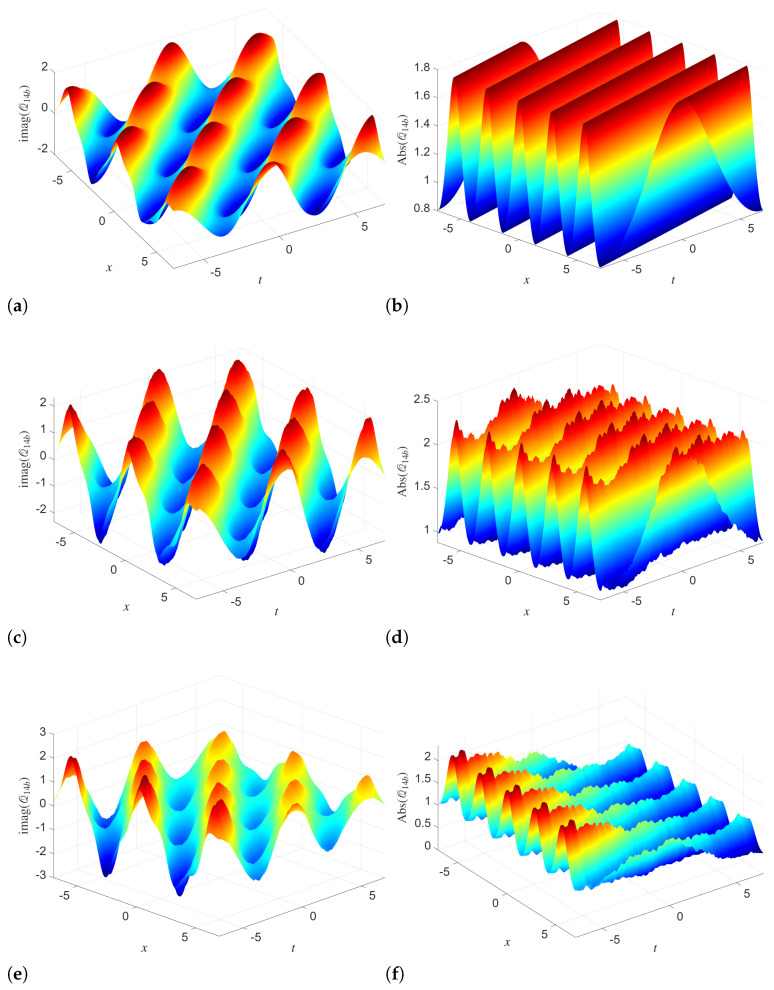
Simulations of the analytical solution $Q_{14a}$ with different noise levels and parameters utilized as $B=2,\;\kappa_{0}=1,\;\kappa_{1}=1,\;\lambda =1,\;\alpha_{1}=1,\;\alpha_{2}=1,\;\gamma_{1}=1,\;\gamma_{2}=0.2$. (**a**) $\sigma_{1}=0$ (**b**) $\sigma_{1}=0$ (**c**) $\sigma_{1}=0.01$ (**d**) $\sigma_{1}=0.01$ (**e**) $\sigma_{1}=0.05$ (**f**) $\sigma_{1}=0.05$.

**Figure 2:**
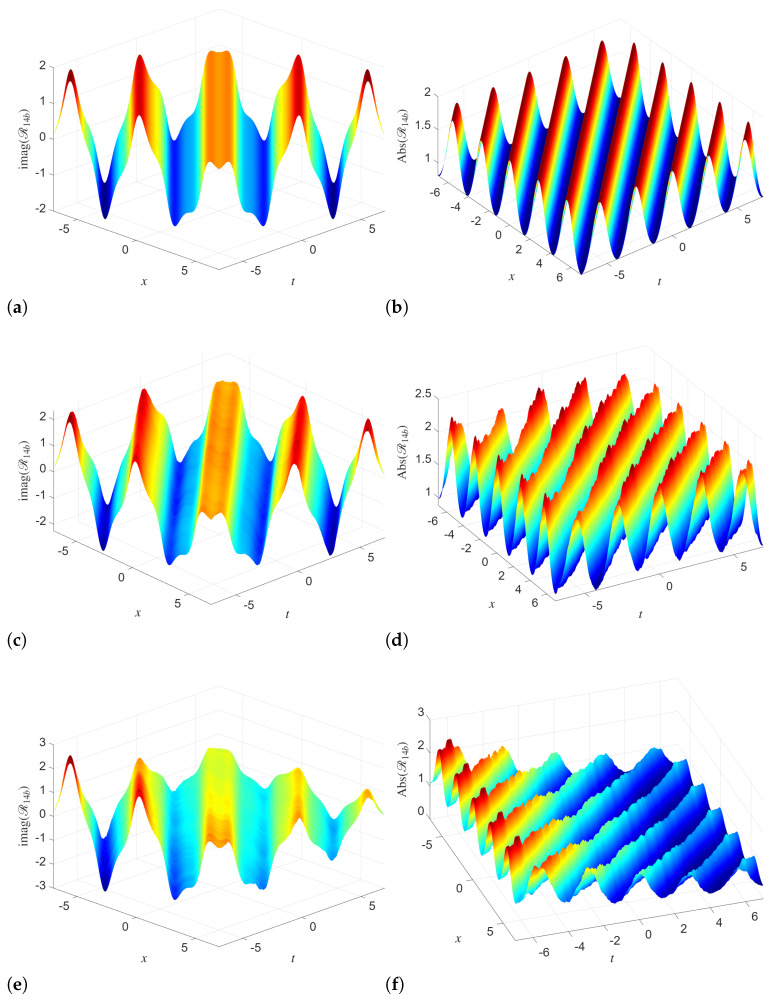
Simulations of the analytical solution $R_{14a}$ with different noise levels and parameters utilized as $B=2,\;\kappa_{0}=1,\;\kappa_{1}=1,\;\lambda =1,\;\alpha_{1}=1,\;\alpha_{2}=1,\;\gamma_{1}=1,\;\gamma_{2}=0.2$. (**a**) $\sigma_{2}=0$ (**b**) $\sigma_{2}=0$ (**c**) $\sigma_{2}=0.01$ (**d**) $\sigma_{2}=0.01$ (**e**) $\sigma_{2}=0.08$ (**f**) $\sigma_{2}=0.08$.

The 3D simulation of solution $Q_{14a}$ in Figure 1 shows the progressive influence of stochasticity on phase and amplitude dynamics. In the deterministic case, when we use $\left(\sigma_{1}=0\right)$, the imaginary part shows smooth and regular wave dynamics. Here, the absolute value remains well-organized with near uniform pattern. When we increase the noise intensity from 0 to $\sigma_{1}=0.01$ and $\sigma_{1}=0.05$, then noticeable distortions appear in the amplitude surface, but the overall shape is preserved. Specifically, the multiplicative noise induces small-scale fluctuations and roughness in the magnitude. This shows the modulation of the underlying coherent structure without completely destroying it. This, in turn, highlights the robustness of the analytical solution under weak to moderate stochastic perturbations. Similar dynamics are observed in the simulations of $R_{14a}$ in Figure 2. For $\sigma_{2}=0$, both the imaginary part and the absolute value display highly regular, periodic waves. As $\sigma_{2}$ increases, the imaginary part remains largely oscillatory, but the amplitude surface becomes increasingly irregular, with visible deformation of the crests and enhanced variability. At high noise levels, the absolute value demonstrates stronger attenuation and loss of symmetry. This shows that the coupled Manakov system responds asymmetrically to noise, where the amplitude is more sensitive than the phase.

**Figure 3:**
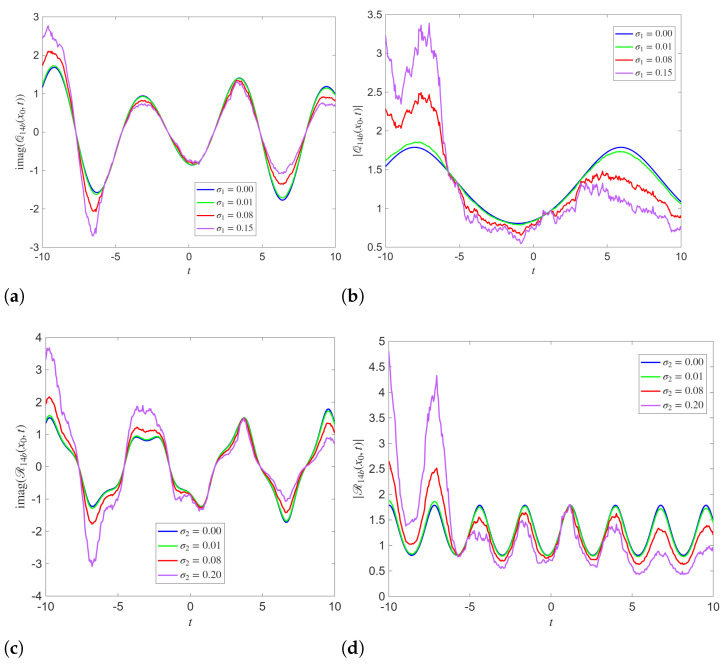
2D Simulations of the analytical solutions $Q_{14a}$ and $R_{14a}$ with different noise levels and x=3 with parameters $B=2,\;\kappa_{0}=1,\;\kappa_{1}=1,\;\lambda =1,\;\alpha_{1}=1,\;\alpha_{2}=1,\;\gamma_{1}=1,\;\gamma_{2}=0.2$. (**a**) $Im(Q_{14b})$ for different $\sigma_{1}$ (**b**) $|Q_{14b}|$ for different $\sigma_{1}$ (**c**) $Im(R_{14b})$ for different $\sigma_{2}$ (**d**) $|R_{14b}|$ for different $\sigma_{2}$.

The 2D dynamics simulated in Figure 3 with varying *t* and fixed x=3 clarify the influence of the noise on system dynamics. For both $Q_{14a}$ and $R_{14a}$, the imaginary parts keep their oscillatory behavior as noise strength increases. But the extrema of these solutions become more pronounced and less smooth. The absolute values depict clear deviations from the deterministic profile. These results confirm that stochasticity amplifies temporal variability and introduces intermittency into the system.

**Figure 4:**
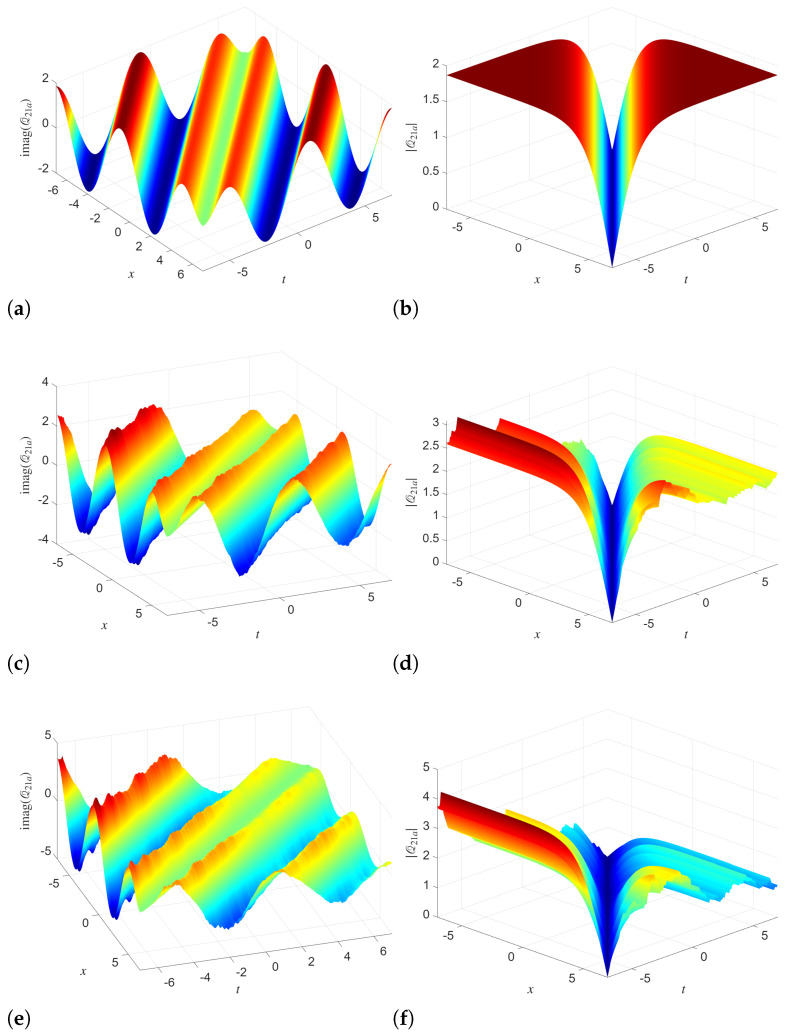
Simulations of the analytical solution $Q_{21a}$ with different noise levels and parameters utilized as $B=2,\;\kappa_{0}=1\;\kappa_{1}=1,\;\lambda =0.01,\;\alpha_{1}=1,\;\alpha_{2}=1,\;\gamma_{1}=1,\;\gamma_{2}=3$. (**a**) $\sigma_{1}=0$ (**b**) $\sigma_{1}=0$ (**c**) $\sigma_{1}=0.1$ (**d**) $\sigma_{1}=0.1$ (**e**) $\sigma_{1}=0.2$ (**f**) $\sigma_{1}=0.2$.

**Figure 5:**
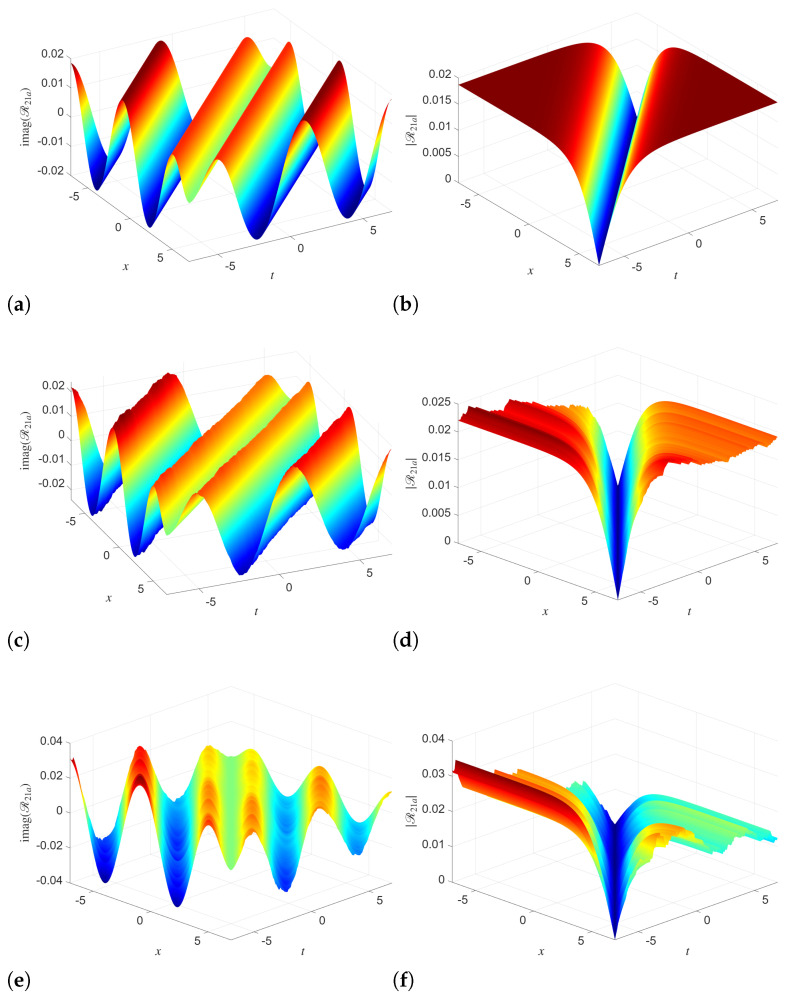
Simulations of the analytical solution $R_{21a}$ with different noise levels and parameters utilized as $B=2,\;\kappa_{0}=1\;\kappa_{1}=1,\;\lambda =0.01,\;\alpha_{1}=1,\;\alpha_{2}=1,\;\gamma_{1}=1,\;\gamma_{2}=3$. (**a**) $\sigma_{2}=0$ (**b**) $\sigma_{2}=0$ (**c**) $\sigma_{2}=0.05$ (**d**) $\sigma_{2}=0.05$ (**e**) $\sigma_{2}=0.15$ (**f**) $\sigma_{2}=0.15$.

The 3D surface plots of the solutions $Q_{21a}$ and $R_{21a}$ are simulated with different noise intensities in Figure 4 and Figure 5, respectively. In the absence of noise, both $Q_{21a}$ and $R_{21a}$ display smooth, well-organized wave patterns in the imaginary part and sharply localized valley structures in the absolute value. With increasing noise strength, the imaginary-part surfaces retain their global oscillatory structure but develop surface roughness and local deformations. The amplitude surfaces experience significant smoothing and broadening of the localized valley, accompanied by visible irregular fluctuations along both spatial and temporal directions.

**Figure 6:**
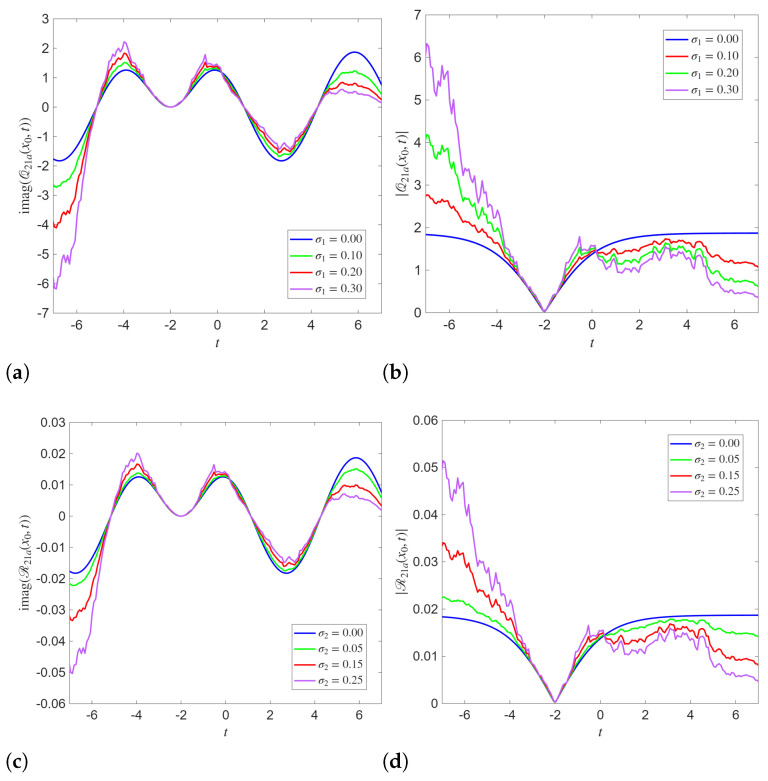
(**a**–**d**) 2D Simulations of the analytical solutions $Q_{21a}$ and $R_{21a}$ with different noise levels and x=3 with parameters $B=2,\;\kappa_{0}=1\;\kappa_{1}=1,\;\lambda =0.01,\;\alpha_{1}=1,\;\alpha_{2}=1,\;\gamma_{1}=1,\;\gamma_{2}=3$.

The 2D profiles of the analytical solutions $Q_{21a}$ and $R_{21a}$, at the fixed spatial point x=3 are demonstrated in Figure 6. These simulations depict the effect of the increasing noise intensity on both phase and amplitude dynamics. In deterministic simulations, the imaginary parts show smooth oscillatory dynamics. The absolute values at $\sigma_{1}=\sigma_{2}=0$ display a clear cusp-like minimum. As noise parameters $\sigma_{1}$ and $\sigma_{2}$ increase, the amplitudes of the imaginary increasingly distort near the extrema. This phenomenon reflects the sensitivity of the phase component to stochastic perturbations. Further, the absolute values of $Q_{21a}$ and $R_{21a}$ demonstrate a stronger response to the noise compared to their imaginary counterparts.

**Figure 7:**
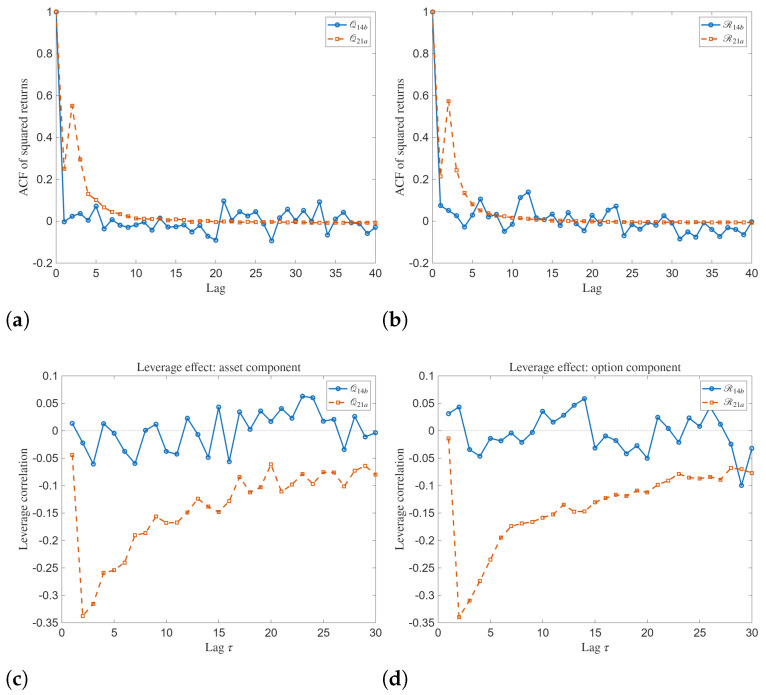
(**a**–**d**) Autocorrelation function (ACF) of square returns and leverage correlation (LC) dynamics of the analytical solutions $Q_{14b}$, $R_{14b}$, $Q_{21a}$ and $R_{21a}$ with x=3.

It should be noted that, for all numerical diagnostics, we use a uniform time grid $t_{k}=k\Delta t$ on [0,T] and generate Wiener increments by

$$W_{j}\left(t_{k+1}\right)=W_{j}\left(t_{k}\right)+\sqrt{\Delta t}\;\xi_{j,k},\;\xi_{j,k}∼N\left(0,1\right),$$

independently for j∈{1,2} and for all *k*. For each realization, the analytical expressions for the solutions are evaluated on the (x,t) grid and the time series $V\left(t\right)=|Q\left(x_{0},t\right)|$ and $\left|R(x_{0},t)\right|$ are extracted at a fixed spatial location $x_{0}$. Returns are computed as $r\left(t_{k}\right)=\Delta \ln\left(V\left(t_{k}\right)+\varepsilon \right)$, and the reported ACF/leverage/PDF and cross-correlation measures are computed from these series. The ACFs of squared returns and LC dynamics for analytical solutions $Q_{14b}$, $R_{14b}$, $Q_{21a}$, and $R_{21a}$ are demonstrated in Figure 7. The ACFs of squared returns for analytical solutions $Q_{14b}$, $R_{14b}$, $Q_{21a}$, and $R_{21a}$ depict distinct differences in second order temporal dependence. The ACF exhibits a strong peak at zero lag, followed by a rapid decay toward values fluctuating around zero as the lag increases. These dynamics show short-range dependence in squared returns and suggest that volatility clustering is present but weak. The ACFs associated with $Q_{14b}$ and $R_{14b}$ show more prominent oscillations around zero at medium lags compared to $Q_{21a}$ and $R_{21a}$.

To support the visual trends, we also report scalar summaries from the computed diagnostics: the zero-lag amplitude correlation Corr(|Q|,|R|) and the peak/initial-lag values of the ACF of squared returns (volatility clustering) for each case.

To complement the PDF plots, we computed the sample kurtosis of the model-generated return series. For the $Q_{14b}$ and $R_{14b}$ cases, the kurtosis values are close to (or below) the Gaussian benchmark 3 (2.965 and 2.544, respectively), indicating approximately Gaussian or sub-Gaussian tails. In contrast, the $Q_{21a}$ and $R_{21a}$ cases exhibit very large kurtosis (105.455 and 121.171), which quantitatively confirms strong intermittency and heavy-tailed fluctuations for this regime.

The LC highlights asymmetries in the temporal evolution of the fluctuations. For both $Q_{14b}$ and $R_{14b}$, the LC oscillates around zero with relatively small magnitude. This indicates a weak and alternating relationship between current returns and future variability. The $Q_{21a}$ and $R_{21a}$ exhibit clearly negative leverage correlations at small lags, which gradually increase toward zero as the lag grows. This signifies an asymmetric response of the system, where negative fluctuations are followed by enhanced variability.

**Figure 8:**
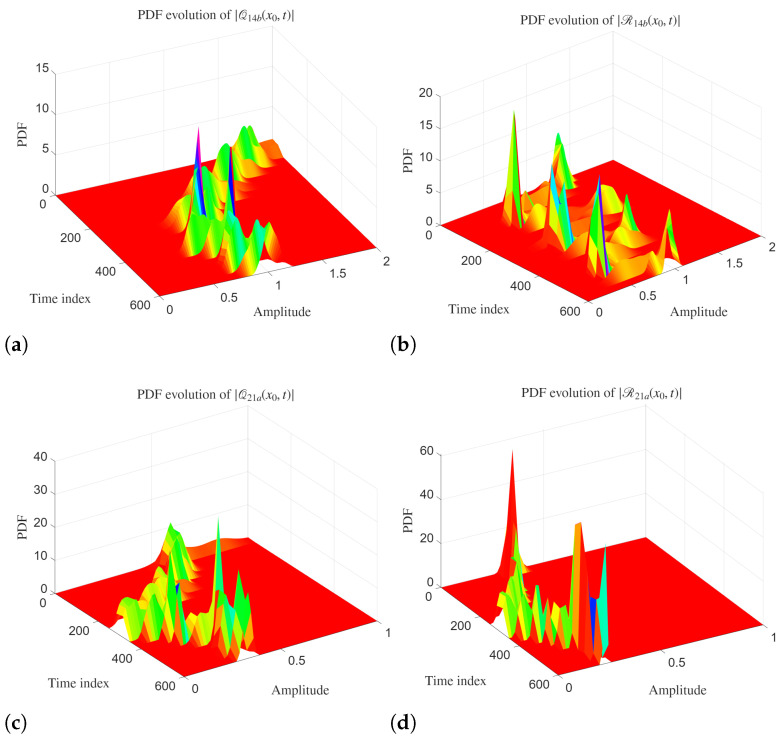
(**a**–**d**) Probability distribution function (PDF) evolution of the analytical solutions $Q_{21a}$ and $R_{21a}$ with x=3.

The PDF evolutions of amplitudes for analytical solutions $Q_{14b}$ and $R_{14b}$ are demonstrated in Figure 8. The PDF evolutions show prominent temporal variability and non-stationary statistical dynamics. The PDFs are highly localized in the amplitude at early times. As the time index increases, we see multiple sharp peaks that disappear intermittently. This shows that the amplitude dynamics are governed by episodic bursts. The presence of multiple peaks demonstrates the impact of the stochastic forcing combined with nonlinear interactions.

For the analytical solutions $Q_{21a}$ and $R_{21a}$, the PDF evolution shows even stronger intermittency and heavier tails. Here, we see that the distributions are concentrated at lower amplitude values. But sporadic large spikes appear at specific times. These extreme peaks are stronger in the $R_{21a}$ case. This shows high susceptibility of this component to stochastic noise.

**Figure 9:**
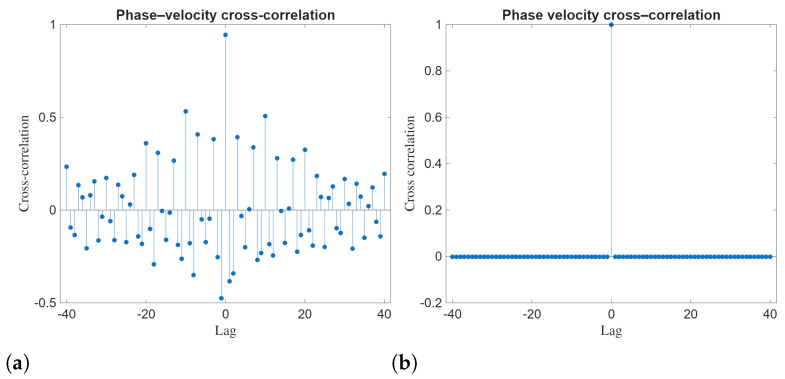
Phase velocity cross correlation of the analytical solutions $Q_{14b}$ vs. $R_{14b}$ and $Q_{21a}$ vs. $R_{21a}$. (**a**) $Q_{14b}$ vs. $R_{14b}$ (**b**) $Q_{21a}$ vs. $R_{21a}$.

The phase-velocity cross-correlation shown in Figure 9 should be interpreted in light of the proportional-component reduction R=λQ. In this polarization-locked subclass, the phases satisfy $\phi_{R}\left(t\right)=\phi_{Q}\left(t\right)+\mathrm{const}$, and therefore the phase velocities coincide, ${\dot{\phi }}_{R}\left(t\right)={\dot{\phi }}_{Q}\left(t\right)$, wherever the phase is well-defined. Consequently, a dominant peak at zero lag is expected. In panel (b), this appears as an (approximately) unit spike at zero lag with negligible values at nonzero lags, confirming near-instantaneous phase-velocity locking.

For panel (a), the small oscillatory values at nonzero lags are attributable to numerical sensitivity in the estimation of phase velocities: (i) phase is ill-conditioned when the instantaneous amplitude becomes very small, and (ii) numerical differentiation amplifies these round-off/unwrap fluctuations. Thus, the nonzero-lag oscillations should not be interpreted as genuine delayed coupling or interaction effects under strict proportionality; rather, Figure 9 primarily serves as a consistency check of phase locking in the proportional-component solutions.

**Figure 10:**
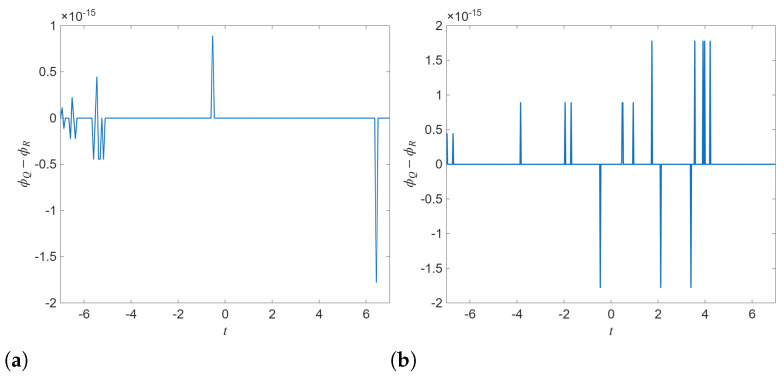
Phase difference of the analytical solutions $Q_{14b}$ vs. $R_{14b}$ and $Q_{21a}$ vs. $R_{21a}$. (**a**) $Q_{14b}$ vs. $R_{14b}$ (**b**) $Q_{21a}$ vs. $R_{21a}$.

The phase-difference curves in Figure 10 are consistent with the proportional-component reduction R=λQ. In particular, the phase difference remains essentially constant (centered near zero) over the entire time interval. The narrow spikes visible in panels (a)–(b) occur at the level of $10^{-15}$ and therefore reflect numerical round-off and phase indeterminacy when the instantaneous amplitude is very small (the phase becomes ill-conditioned near zeros of Q or R), rather than genuine phase-slip or phase-transition events. Consequently, Figure 10 should be interpreted as confirming persistent phase locking in this polarization-locked subclass; nontrivial phase-slip dynamics would require non-proportional (vector) solutions beyond the present reduction.

**Figure 11:**
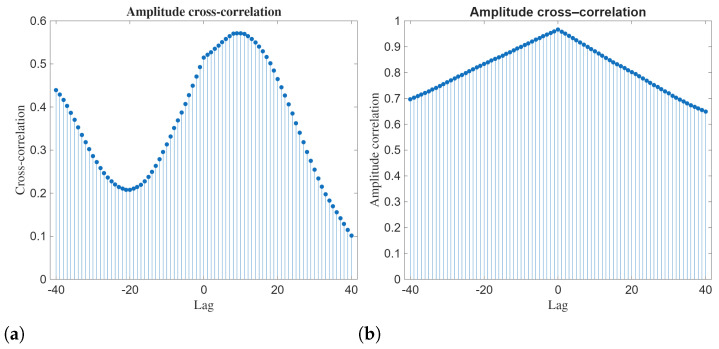
Amplitude cross-correlation of the analytical solutions $Q_{14b}$ vs. $R_{14b}$ and $Q_{21a}$ vs. $R_{21a}$. (**a**) $Q_{14b}$ vs. $R_{14b}$ (**b**) $Q_{21a}$ vs. $R_{21a}$.

The amplitude cross-correlation functions demonstrated in Figure 11 provide information about the strength and structure of the coupling between the two components of the Manakov system. For $Q_{14b}$ and $R_{14b}$, the cross-correlation shows a smooth, asymmetric profile with a clear maximum at a positive lag and a gradual decay toward larger lags. This shows that the amplitude of one component influences the other with a finite delay. The nonuniform shape of the curve shows the presence of oscillatory modulation and delayed response.

The amplitude cross-correlation for $Q_{21a}$ and $R_{21a}$ shows nearly symmetric triangular structure centered at zero lag. Here, we see that the correlation values are high over the entire lag range. The maximum value at zero lag shows strong instantaneous amplitude synchronization. The slow decay away from zero shows long-range coherence in amplitude dynamics. Such dynamics are consistent with the localized and strongly coupled nature of the 21a solution family.

**Figure 12:**
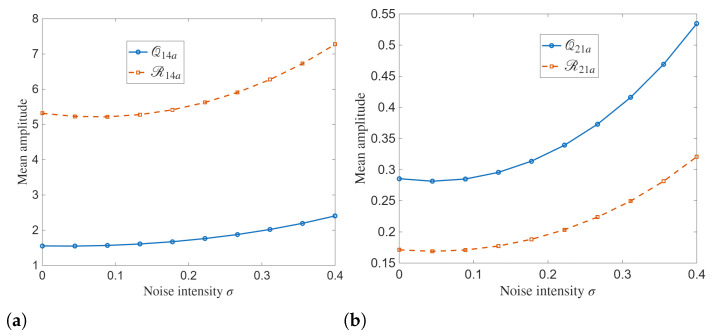
Mean amplitude dynamics of the analytical solutions: (**a**) $Q_{14a}$ and $R_{14a}$ (**b**) $Q_{21a}$ and $R_{21a}$.

The mean amplitude dynamics is demonstrated in Figure 12 as a function of noise intensity σ. This figure reveals the systematic influence of stochastic forcing on the energy of the analytical solutions. For the 14a solution family, both $Q_{14a}$ and $R_{14a}$ show gradual increase in mean amplitude as σ increases. While the deterministic case exhibits nearly constant mean levels, the consistently larger mean amplitude of $R_{14a}$ compared to $Q_{14a}$ shows asymmetry in the coupled components, where the second component is accumulating more energy under stochastic excitation.

A similar growth pattern can be seen in simulations of the 21a solution family. The mean amplitude of $Q_{21a}$ rises rapidly with noise intensity as compared to $R_{21a}$. This behavior shows stronger sensitivity of the first component to stochastic perturbations. The smooth, convex nature of the curves shows that the noise contribution becomes increasingly effective at higher intensities.

**Figure 13:**
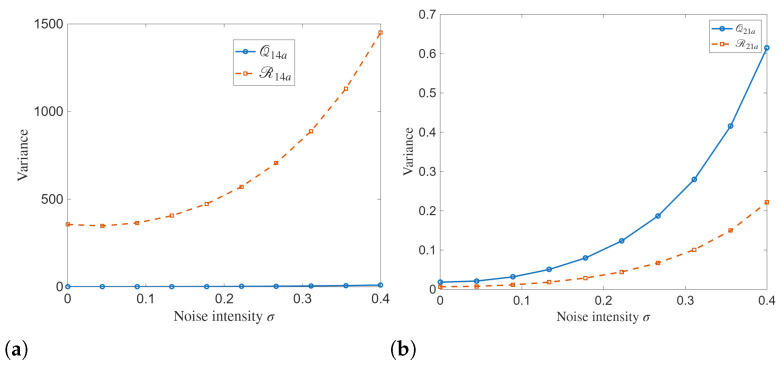
Variance of the analytical solutions: (**a**) $Q_{14a}$ and $R_{14a}$ (**b**) $Q_{21a}$ and $R_{21a}$.

The variance of analytical solutions as a function of noise intensity σ simulated in Figure 13 provides a quantitative measure of stochastic dispersion in the proposed model. For the 14a solution family, the variance of $Q_{14a}$ is small across the entire noise range, which increases only when σ grows. The variance of $R_{14a}$ increases rapidly and nonlinearly with noise intensity. This disparity indicates a strong asymmetry in the sensitivity of the two coupled components.

For the 21a solution family, both $Q_{21a}$ and $R_{21a}$ show monotonic growth in variance as the noise increases. Compared to the 14a case, the variance levels are much smaller, reflecting the more localized and lower-amplitude nature of these solutions.

## 5. Conclusions

We have studied analytically and statistically the Manakov system (MS) under white noise. The analytical investigation has been performed by using the KM approach to derive new solutions for the MS under white noise. These solutions are expressed by Jacobi-elliptic functions, trigonometric functions, hyperbolic functions, and exponential functions, which have not been explored previously in the literature for the considered system. Some statistical investigations have been conducted to illustrate distinct features of the stochastic behaviors of the solutions. All the results have been graphically demonstrated to show the deterministic and stochastic propagations of waves under different values of noise intensities.

The graphs illustrate that the analytical results of the stochastic Manakov system show a balance between robustness and sensitivity to noise. While the core wave structures persist even at higher noise levels, stochastic perturbations greatly affect amplitude modulation and smoothness. Also, graphical analysis predicts that the derived solutions of the stochastic MS exhibit localized waves such as periodic solitons, dark solitons, multi-hump solitons, and cusp-type amplitude structures that are flexible to weak noise but gradually distorted under stronger noise parameters $\sigma_{1}$ and $\sigma_{2}$.

From statistical analysis, the PDF dynamics show the complex stochastic nature of the MS, where multiplicative noises $\sigma_{1}$ and $\sigma_{2}$ and nonlinear coupling generate non-Gaussian, time-dependent distributions identified by variation and extreme-event dominance. The combination of near–zero phase differences and distinct cross-correlation graphs shows that distinct soliton solution classes of the stochastic MS encode fundamentally different inter-component coupling mechanisms. The ACF for specific soliton solutions show strong peak at zero lag, followed by a rapid decay toward a variation of values around zero as the lag increases, which display short range dependence in squared returns and predicts that volatility clustering is present but weak. The LC shows asymmetries in the temporal evolution of the fluctuations. These outcomes show that noise intensity $\sigma_{1}$ and $\sigma_{2}$ play a vital role in controlling fluctuation strength and stability of the acquired soliton solutions. While some solution components remain relatively stable even under strong noise, others undergo rapid variance growth, signaling enhanced instability and dispersion.

A natural extension of the present analysis is to allow an adaptive (state-dependent) market-heat potential instead of the constant choice A(ζ,y)=ζ, and to investigate how adaptation modifies the obtained wave families and stochastic diagnostics.

## Data Availability

Not applicable.
